# Structural imbalances in EPA Perceptions: A multi-center study of cognitive differences between faculty and trainees in Chinese surgical residency training

**DOI:** 10.1016/j.sopen.2026.06.003

**Published:** 2026-06-26

**Authors:** Yuan Xu, Ling Wang, Mao Mu, Haiyang Li

**Affiliations:** a Department of Surgery, Clinical Medical College, Guizhou Medical University, Guiyang, China; b Department of Medical Quality and Safety Management, Affiliated Hospital of Guizhou Medical University, Guiyang, China; c Clinical Medical College of Guizhou Medical University, Guiyang, China

**Keywords:** Entrustable Professional Activities (EPA), Surgical Residency Training, Cognitive Differences, Behavioral Anchors

## Abstract

**Objective:**

This study aimed to identify and quantify systematic cognitive differences between clinical faculty and surgical residents regarding core competency requirements in standardized residency training, using the Entrustable Professional Activities (EPA) framework, and to provide evidence for optimizing competency-based training curricula.

**Methods:**

A multicenter cross-sectional anonymous online questionnaire survey was administered in March 2025 to 42 supervising faculty and 68 surgical residents across three training bases in Guizhou Province. The questionnaire was developed in accordance with the 2022 Edition of the National Standards and Contents for Residency Training in China, covering 100 EPA items across five subspecialties: neurosurgery, general surgery, urology, orthopedics, and anesthesiology. Participants rated the importance and necessity of each item using a 4-point Likert scale. Reliability was evaluated using Cronbach's α and intraclass correlation coefficient (ICC). Group differences were analyzed via independent-samples Mann–Whitney *U* tests, with Cohen's d for effect size, and inter-rater consistency was assessed using Kendall's W.

**Results:**

A total of 110 valid questionnaires were recovered (valid response rate: 93.2%). The overall Cronbach's α was 0.951, and the test–retest ICC was 0.89, indicating excellent reliability. High consistency (> 90%) was observed between faculty and residents for general surgery and neurosurgery EPAs. Among 19 orthopedic EPAs, 10 items showed significant differences (*P* < 0.05), with Cohen's d ranging from 0.82 to 1.55; residents consistently rated these items higher than faculty. For three anesthesiology items related to “Life Monitoring and Resuscitation,” faculty scored a mean of 4.00 ± 0, whereas residents scored 3.36 ± 1.03 (d = 0.98, *P* < 0.01). Kendall's W ranged from 0.10 to 0.17, indicating weak overall consistency. Both groups emphasized strengthening training in soft skills, including medical documentation and physician–patient communication.

**Conclusion:**

Systematic discrepancies exist between faculty and residents in perceptions of orthopedic procedural skills and anesthetic safety during surgical residency. To address this gap, unified entrustment benchmarks, integrated assessable soft-skill training, and continuous formative feedback should be prioritized to improve competency-based surgical residency education.

Standardized training is the key method for medical trainees to become clinical physicians. The EPA concept was first proposed by Professor Olle ten Cate from Utrecht University in the Netherlands in 2005. Its core definition is: “A delegable professional activity refers to those professional practice units that can be entrusted to trainees for independent execution once they have demonstrated the required competencies.” [1]. In the highly practical discipline of surgery, the Entrustable Professional Activities (EPA) framework plays a critical role by transforming training objectives into a series of clinical tasks that can be gradually authorized, thereby providing a clear path for competency-oriented training [2]. Surgical residency training covers clinical and operative skills across multiple specialties [3]. In China, the standardized training period for surgical residents is 36 months. During this period, students are required to complete the training content related to general surgery, neurosurgery, urology, orthopedics, and anesthesiology. The Chinese Medical Doctors Association solicited opinions from experts in various fields, health administrative departments at all levels, relevant training bases, guiding physicians, and trainees. They received 3000 pieces of feedback. The Chinese Medical Doctors Association classified and sorted these opinions, and adopted 610 reasonable suggestions, forming the “Standardized Training Content for Resident Physicians (2022 Edition)” for the training. At the beginning of our research, this complete training content and training standards had been applied for 3 years.

In this context, EPA serve as an assessment framework centered on real clinical tasks, highlighting the need for instructors to gradually authorize trainees to perform tasks independently based on their competency levels [4]. However, inconsistencies in understanding training objectives and core competencies between Faculty and trainees, as well as varying perceptions related to seniority, may reduce trainees' motivation and engagement, negatively affecting their commitment and the overall quality of training [5], [6]. The construction of surgical EPA includes multiple aspects: routine skills aligned with the curriculum, key surgical operations in senior years, and often overlooked soft skills such as doctor-patient communication and medical humanities. The detailed training requirements for surgery in the “Regulatory Training Curriculum for Resident Physicians (2022 Edition)” stipulate that during the training period, one must master the pathogenesis, clinical characteristics, diagnostic and differential diagnostic key points, and treatment principles of various common and frequently-occurring diseases. At the same time, the number of patients managed by the trainees, as well as the number of operations and surgeries they participate in, all need to meet the corresponding requirements (Attachment 1 and Attachment 2). This approach aims to systematically enhance the quality of resident training and implement authorized teaching [7], [8]. The operationalization of these non-technical domains within EPA frameworks remains underexplored, particularly in resource-constrained settings.

However, in China's surgical residency training, few studies have systematically and quantitatively examined cognitive differences between teachers and students across multiple centers regarding EPA. During the training process, the trainees are more eager to quickly master a skill that can be independently accomplished; in contrast, the teachers will pay more attention to whether this project can ensure the safety of the patients. Such a cognitive difference is not conducive to achieving a satisfactory outcome for both the teachers and the trainees. In order to better analyze the cognitive differences between the teachers and the trainees and understand the viewpoints of all the training content, we conducted this study.

## Objects and methods

### Survey subjects

In March 2025, a total of 110 Faculty and trainees from three home training bases in Guizhou Province were selected as survey subjects. Among them, 42 Faculty were all from the Affiliated Hospital of Guizhou Medical University. A total of 68 trainees were randomly selected from the surgical resident trainees of the Affiliated Hospital of Guizhou Medical University, Guizhou Provincial People's Hospital, and Bijie Hospital of Zhejiang Provincial People's Hospital. Before training, the trainees came from six hospitals across provincial, municipal, and county levels.

Among the survey subjects, all the trainees were resident doctors. They were either currently undergoing standardized training for resident physicians or had completed it within the past three years. All the Faculty are senior attending physicians or above who have completed at least three years of standardized residency training and have been involved in teaching this training for a long time. This ensures their familiarity with the residency training content and guarantees the authenticity and validity of the completed questionnaires.

### Investigation methods

This study was based on the content and standards of standardized training for resident physicians (2022 Edition) issued by the Chinese Medical Doctor Association. According to the training project content of this standard, a questionnaire is designed and distributed online to Faculty and trainees at the surgery resident training base. The informed consent of all participants in the study has been obtained. During the sample collection process, personal identification information will be used for processing to protect the privacy of the participants. The data analysts will only see the individual identification numbers and will no longer access the participants' names, phone numbers, etc.

### Investigation contents

(1) Basic information: including identity as teacher or student, work and training units, and years of participation in standardized training for resident physicians; (2) Specialized field: general surgery, urology, neurosurgery, anesthesiology, cardiothoracic surgery, and other surgical residency programs; (3) Training content: whether trainees in the general surgery major master all required contents during rotations in related departments, consistent with actual work requirements; (4) Evaluation criteria: A 4-point scoring system was adopted. Participants rate training content based on importance, frequency, and consequences as follows:

4 points - Extremely important: tasks frequently encountered in daily work; failure to perform correctly or timely may cause patient death or permanent disability.

3 points - Important: tasks occur at least once a week; errors may cause serious patient harm.

2 points - Somewhat important: tasks rarely encountered (less than once a month).

1 point - Irrelevant, unimportant, or unreasonable.

### Quality control

The investigators consulted experts and reviewed relevant literature when designing the questionnaire, and then revised it based on feedback to ensure scientific rigor. Reliability analysis and testing were conducted on the results of the questionnaire survey. The investigators used the online platform “Wenjuanxing” to create the questionnaires. The respondents completed and submitted the questionnaires under their real names via WeChat to complete the survey. Each wechat account could only fill in the questionnaires once.

Criteria for determining invalid questionnaires: (1) Not filled out under real name; (2) The questionnaire items are not filled out completely; (3) All project scoring options are consistent. If any of the above three items is met, the questionnaire will be deemed invalid.

### Statistical methods

The results of the online questionnaire survey were directly exported from the database and statistically analyzed using SPSS 26.0. Normally distributed continuous variables were expressed as mean ± standard deviation (mean ± SD), and Mann-Whitney *U* tests were used for group comparisons with a two-sided significance level of α = 0.05.

## Results

### General overview

We distributed 118 questionnaires to Faculty and trainees participating in standardized training for resident physicians. We excluded 8 invalid questionnaires due to incomplete responses and a small number of participants. A total of 110 valid questionnaires were collected, including 42 from Faculty and 68 from trainees (Table 1). For Faculty, the years of standardized training were calculated from the date they obtained the resident physician training teacher qualification certificate until the study date. For trainees, years were calculated from their first year of standardized resident physician training. Most standardized training Faculty come from provincial tertiary hospitals, while the trainees come from various hospital levels, including provincial hospitals (58.82%), municipal hospitals (30.89%), county-level hospitals (10.29%), and other hospital types. In terms of the years of entering standardized training, there is a significant difference between teachers and trainees, indicating that the teaching staff are mainly senior physicians, whereas the trainees are at an early stage of training. This difference in experience can explain the difference in cognition.

**Table 1:** Basic demographic and professional characteristics of the participants.

Characteristic	Overall (*n* = 110)	Faculty (*n* = 42)	Trainees (*n* = 68)
Hospital level			
Provincial	82 (74.55%)	42 (100%)	40 (58.82%)
Municipal	21 (19.09%)	0	21 (30.88%)
County	7 (6.36%)	0	7 (10.29%)
Professional title			
Senior	66 (60.00%)	27 (64.29%)	0
Intermediate	17 (15.45%)	15 (35.71%)	2 (2.94%)
Junior and below	27 (24.55%)	0	66 (97.06%)
Specialty			
Neurosurgery	14 (12.73%)	3 (7.14%)	11 (16.18%)
General Surgery	22 (20.00%)	7 (16.67%)	15 (22.06%)
Urology	20 (18.18%)	5 (11.90%)	15 (22.06%)
Anesthesiology	27 (24.55%)	16 (38.10%)	11 (16.18%)
Orthopedics	27 (24.55%)	11 (26.19%)	16 (23.53%)
Years in standardized training, mean ± SD	3.86 ± 3.244	5.976 ± 3.942	2.456 ± 1.332

### Reliability analysis and consistency test

We distributed a survey on the importance and necessity of surgical standardized training content to 118 Faculty and trainees, and retrieved 110 valid questionnaires. The Cronbach's alpha coefficient for all questionnaires exceeded 0.9, indicating high reliability of the questionnaire design and responses.(Table 2).

**Table 2:** Reliability analysis of the survey questionnaire across departments.

Department	Cronbach's α	Standardized Cronbach's α	Number of Items
Neurosurgery	0.937	0.941	16
General Surgery	0.951	0.955	24
Urology	0.955	0.957	22
Anesthesiology	0.958	0.960	19
Orthopedics	0.949	0.950	19

Kendall's W analysis was used to test the consistency of evaluations in this questionnaire survey. (Table 3) The levels of training content across all specialties show statistically significant differences, indicating variation among specialties. However, Kendall's W coefficients were all below 0.2, indicating a low degree of agreement among evaluators. This suggests that although standardized training content covers many topics across specialties, evaluators differ in their assessments of importance.

**Table 3:** Results of the consistency test (Kendall's W).

Department	Kendall's W coefficient	χ^2^	*p*-value
Orthopedics	0.102	49.62	<0.001***
Neurosurgery	0.141	29.529	0.014**
General Surgery	0.106	53.393	<0.001***
Urology	0.165	69.397	<0.001***
Anesthesiology	0.163	78.989	<0.001***

### Differences in the understanding of training content between resident training faculty and trainees

To clarify whether cognitive differences exist between Faculty and trainees about the training program content, we analyzed evaluations from both groups. We found that their perceptions of the training content differed across various professional fields. Specifically, opinions on the importance of training content in general surgery and neurosurgery were relatively consistent, with agreement rates of 91.67% and 93.75%, respectively. In contrast, there was significant disagreement regarding orthopedic training content, as the agreement rate dropped to 47.37%.

As visually summarized in Table 4, significant perception gaps are concentrated in procedural skills in orthopedics and safety-critical competencies in anesthesiology.

**Table 4:** Differences in the perception of training program importance between faculty and trainees.

	Total number of training programs	The cognitive differences between teachers and students	Proportion of different items
Orthopedics	19	10	52.63%
Anesthesiology Department	19	3	15.79%
Urology Department	22	6	27.27%
General Surgery Department	24	2	8.33%
Neurosurgery Department	16	1	6.25%

The training content covers specific operations of common orthopedic treatment techniques, such as splints, plaster casts, and bone traction fixation. It also includes the significance, operational methods, and prevention and management of complications related to closed therapy. The training covers common fractures and dislocations, diagnostic examinations for bone tumors, and related imaging and laboratory tests. Furthermore, the training involves common treatment methods and surgical techniques for orthopedic trauma, mainly fractures and dislocations. Additional topics include debridement of hand injuries, repair of skin defects, and basic techniques such as tendon anastomosis and orthopedic internal fixation. The training also covers non-surgical treatments and principles for various conditions, including lumbar intervertebral disc protrusion, cervical spondylosis, and lumbar sprain, as well as stenosing tenosynovitis, meniscus injury, and tennis elbow. Specialists believe that the degree of correlation with the standardized training of general surgery is not high. However, trainees had a relatively high recognition of the importance of the relevant content (*P* < 0.05). This reflects the trainees' renewed concern and interest in clinical operational and applied skills (Table 5). The situation in anesthesiology training is quite different. Faculty rated the importance of non-invasive monitoring techniques—such as electrocardiogram, blood pressure, pulse, respiration, and body temperature—and cardiopulmonary and cerebral resuscitation techniques consistently at 4 points. However, trainees gave significantly lower recognition scores of 3.36 and 3.04, respectively (*P* < 0.05). It is hypothesized that trainees who participated in the survey encountered fewer rescue cases, such as cardiopulmonary resuscitation, during their training. This may have led to insufficient understanding and awareness of intraoperative monitoring and resuscitation techniques.

**Table 5:** Key areas of perception difference regarding training importance between Faculty and trainees.

Training content (item)	Faculty (Mean ± SD)	Trainees (Mean ± SD)	Statistic	*p*-value	Cohen's *d*
Orthopedics (10/19)					
Hands-on techniques (splinting, casting, skeletal traction)	3.09 ± 0.94	3.75 ± 0.58	53.5	0.041*	0.88
Therapeutic nerve block: principles, technique, and complication management	2.55 ± 0.69	3.44 ± 0.89	36.0	0.007**	1.09
Common fractures and dislocations	3.27 ± 0.79	3.88 ± 0.34	49.0	0.016*	1.07
Physical examination for bone tumors	2.46 ± 0.69	3.68 ± 0.60	21.0	< 0.001***	1.93
Imaging and laboratory investigations in orthopedics	3.18 ± 0.75	3.81 ± 0.40	45.5	0.014*	1.11
Management and surgical techniques for orthopedic trauma	3.09 ± 0.94	3.88 ± 0.34	47.0	0.012*	1.20
Debridement of hand trauma	3.09 ± 1.14	3.81 ± 0.40	53.5	0.040*	0.92
Management of skin defects	2.82 ± 1.08	3.75 ± 0.45	40.0	0.008**	1.22
Basic techniques for tendon repair and internal fixation	2.91 ± 0.70	3.75 ± 0.58	32.5	0.002**	1.34
Non-operative management for common conditions (e.g., LDH, cervical spondylosis)	2.73 ± 0.65	3.69 ± 0.60	27.0	0.001**	1.55

Anesthesiology (3/19)					
Non-invasive monitoring (ECG, NIBP, pulse, respiration, temperature)	4.00 ± 0.00	3.36 ± 1.03	120.0	0.011*	0.98
Cardiopulmonary cerebral resuscitation (CPCR)	4.00 ± 0.00	3.45 ± 1.04	112.0	0.030*	0.83
Anesthesia-surgery coordination	3.94 ± 0.25	3.27 ± 0.79	131.5	0.005**	1.25

Urology (6/22)					
Application of urological imaging (KUB, CT, MR, etc.)	3.20 ± 0.45	3.73 ± 0.46	17.5	0.040*	1.17
Percutaneous nephrolithotomy (PCNL)	2.60 ± 0.89	3.60 ± 0.63	14.5	0.026*	1.43
Ureterorenoscopy (URS)	3.00 ± 0.71	3.67 ± 0.90	17.5	0.047*	1.23
Laparoscopic surgery	2.60 ± 0.90	3.60 ± 0.51	13.5	0.022*	1.63
Principles and techniques of intracavitary thermotherapy	1.80 ± 0.84	3.40 ± 0.63	5.5	0.003**	2.34
Urodynamic studies	2.80 ± 0.84	3.53 ± 0.64	18.5	0.069	1.07

General Surgery (2/24)					
Fundamentals of laparoscopic surgery	3.43 ± 0.54	3.80 ± 0.41	33.0	0.089	0.82
Venesection/Venotomy	3.00 ± 0.82	3.67 ± 0.62	27.5	0.045*	0.98

Neurosurgery (1/16)					
Pathogenesis of common neurosurgical diseases	2.33 ± 0.58	3.36 ± 0.67	4.5	0.045*	1.56

### Opinions and suggestions from resident training Faculty and trainees regarding the training content

This study collected suggestions from Faculty and trainees via questionnaires to improve the EPA training content in surgery. The feedback from respondents indicates that the training content for each subject still requires targeted strengthening. Many Faculty pointed out that the standardization of medical record writing, doctor-patient communication, and humanistic care are core capabilities for medical trainees transitioning into clinical physicians. They suggested integrating this training throughout the entire training process. This indicates that the current resident training system in our country needs to further improve and enforce detailed rules to cultivate the above-mentioned capabilities.(Table 6.)

**Table 6:** Suggested additional training content by Faculty and trainees.

Specialty	Theoretical training needs	Practical skills training needs
Thoracic Surgery	Pathophysiology of pneumothorax, Comprehensive lung cancer therapy, Theory of pulmonary bullae	Chest tube removal, Case collection on pulmonary bullae
Neurosurgery	Neuro-critical care management, Management of acute cerebral hemorrhage and infarction, Theory of cerebral hemorrhage	Neurological physical examination, Simulated surgical procedures
General Surgery	Surgical principles for malignant tumors, Medical documentation standards	Sentinel lymph node biopsy for breast cancer (≥5 cases), Surgery for perianal abscess and hemorrhoids, Aseptic draping and prepping procedures
Urology	Adrenal gland diseases and tumors, Urosepsis	Circumcision, Case collection on adrenal disorders (1–2 cases)
Anesthesiology	Adverse drug reactions in anesthesia, ERAS protocols, Principles of vasoactive drugs	Spinal anesthesia/Central venous catheterization, Laryngeal mask airway insertion, Ultrasound-guided techniques, Preoperative patient consultation and communication
Orthopedics	Mechanisms of spinal cord injury, Principles of bone tumor treatment, Medical image interpretation	Complex wound dressing change, Rehabilitation techniques, Physical examination training

## Discussion

This study employed a questionnaire survey to assess Faculty and trainee perceptions of Entrustable Professional Activities (EPAs) in surgical residency training. It revealed systematic cognitive discrepancies across core specialties—particularly in orthopedics, anesthesiology, and urology—suggesting a structural imbalance in how training priorities are allocated within the EPA framework. Specifically, trainees assigned higher importance to procedural skills (e.g., fracture fixation, wound debridement), reflecting their eagerness for hands-on experience and readiness for advanced entrustment. In contrast, Faculty prioritized safety-critical, foundational competencies such as intraoperative monitoring and resuscitation, underscoring their risk-averse stance shaped by clinical experience. [9], [10] The key point is that this disparity can be interpreted from the perspective of “authorization gradient”: Although the trainees expect to have greater autonomy and more practical operation opportunities, teachers always impose stricter authorization conditions due to basic safety considerations, limiting the degree of mastery of the tasks. Furthermore, both groups emphasized the need to strengthen “soft skills” (e.g., doctor–patient communication, medical documentation), indicating that the current EPA implementation remains skewed toward technical proficiency at the expense of humanistic and integrative competencies—another dimension of structural imbalance in competency design.

This study found that the consistency between Faculty and trainees in evaluating the importance of most EPA items was relatively weak (Kendall's W = 0.10–0.17). This finding reflects the absence of a unified behavioral observation standard and a standardized judgment anchor in the current EPA implementation. [11], [12], [13], [14] For example, behaviorally anchored rating scales (BARS) are not yet employed. Although the “Contents and Standards of Standardized Training for Resident Physicians (2022 Edition)” introduced the competency-oriented concept, a specialized and reliable behaviorally anchored rating scale (BARS) for professional activities has not yet been developed. International experience shows that effective EPA authorization depends on specific behavioral indicators. These indicators clearly specify the circumstances, performance levels, and responsibilities eligible for delegation [9], [16], [17]. Without such operational definitions, Faculty often delegate authority subjectively. Meanwhile, trainees struggle to understand how to improve their abilities. Therefore, it is urgently necessary to combine the clinical reality in China, construct an EPA behavioral description library covering all subspecialties of surgery, and embed formative assessment tools to bridge the gap between “standards and practices”.

Faculty and trainees both emphasize the need to cultivate “soft skills” such as medical record writing and doctor-patient communication. As a surgeon, the trainees' understanding and hands-on participation in the anesthesia management during the surgical procedures will be of great help in the growth of a surgical resident [18]. However, if it merely stays at the level of advocating concepts, it is difficult to incorporate it into the current assessment system. Therefore, the international medical education community has turned to making such capabilities “explicit” as observable and evaluable sub-tasks of Entrustable Professional Activities. For instance, “effective communication” can be broken down into specific clinical scenarios such as “independently completing preoperative informed consent conversations”, “communicating poor prognosis to family members”, and “guiding patients to self-manage after discharge”. Structured scoring was carried out in conjunction with frameworks such as LCSAS (Liverpool Communication Skills Assessment Scale) or SEGUE [19], [20]. Structured onboarding programs for International Medical Graduates (IMGs) in the Middle East–featuring centralized communication, independent assessors, workload management, and digitization–have demonstrated high satisfaction and documented competency gains. [21]. In the future, common soft skills scenarios in surgery should be systematically sorted out, localized assessment tools should be developed, and they should be integrated into daily teaching activities (such as teaching ward rounds and case reports), so as to transform abstract literacy into specific ability units that can be taught, practiced and evaluated.

In conclusion, we investigate the results confirmed significant differences in the understanding of core EPAs between Faculty and trainees. These differences were consistent across surgical resident training. Currently, research on China's standardized training system for resident doctors focuses mainly on innovating teaching methods. However, studies on changes in teaching content amid the high-quality development of medical education remain limited [4], [22], [23], [24], [26]. Optimizing the current standardized training system for resident doctors should go beyond adjusting content or teaching methods; it should focus more on building consensus between Faculty and trainees. This study has limitations such as a cross-sectional design and regional sample. In the future, these findings can be further verified by increasing the sample size, conducting longitudinal [16] observations of the training effects, and observing the research in multiple training bases. The issue of cognitive differences revealed in this study is precisely in line with the direction of the connotation construction of resident training promoted at the national level. The “Content and Standards for Standardized Training of Resident Physicians (2022 Edition)” clearly states the “Six core Competencies”, among which “communication and cooperation”, “Professional spirit”, and “patient care” all involve a large number of non-technical capabilities [27]. Although current policies emphasize integrating soft skills such as humanistic care, teamwork, and clinical decision-making throughout the training process and recommend establishing a multi-dimensional evaluation mechanism, training still tends to emphasize operation over communication and results over process. [28], [29] Each year, the National Health Commission of China inspects teaching bases for standardized residency training. However, there is no well-established evaluation framework for assessing communication, consensus, and interactive engagement between faculty and residents regarding teaching content. Such divergent perceptions between instructors and trainees regarding training priorities frequently result in residents' concerns over insufficient and ineffective clinical training. Our empirical data provide quantitative evidence of these structural imbalances, offering concrete implications: (1) policy makers and accreditation bodies(National Health Commission and Chinese Medical Association) should increase the weight of non-technical skill EPAs in base evaluations; (2) clinical supervisor should implement Faculty development programs to standardize understanding of EPA behavioral anchors; and (3)Chinese Medical Doctor Association should design targeted teaching modules for high-discrepancy EPAs—such as orthopedic procedural skills and anesthesia safety-critical skills—to align perceptions between supervisors and trainees and support comprehensive competency growth.

## CRediT authorship contribution statement

**Yuan Xu:** Writing – review & editing, Writing – original draft, Funding acquisition, Conceptualization. **Ling Wang:** Data curation. **Mao Mu:** Project administration. **Haiyang Li:** Supervision, Formal analysis.

## Ethical approval and informed consent

This study was submitted to the ethics committee in September 2025 and was approved. Ethical approval document from the Ethical Committee of Guizhou Medical University. Approval Number: 2026-1. Informed consent forms from all participants have been obtained in this study.

Publication Consent: All participants unanimously agreed to publish the anonymous results of the questionnaire study.

## Human ethics

This study was conducted in accordance with the Declaration of Helsinki Human Ethics and Consent to Participate declarations: This research was submitted to the ethics committee in September 2025 and was approved. The Ethics Committee of GuiZhou Medical University Ethics Approval Document. Approval Number: 2026 lunshen No. (1).All the respondents to the questionnaire agreed to participate in this study.

## Funding

This work was supported by the “2023 Medical Education Research Project” (2023B081) of the Medical Education Branch of the 10.13039/501100006765Chinese Medical Association and the National Medical Education Development Center. This is supported by the “2025 Research Education and Research Feedback to Teaching Funding Project - Teaching Research Project” (JXYJY-2025-034) of the Affiliated Hospital of 10.13039/501100010265Guizhou Medical University.

## Declaration of competing interest

The authors declare no conflicts of interest.

## Data Availability

The data and materials can be obtained by contacting the corresponding author. (If the article is officially published, the data can be included in the supplementary materials of the article.)
