# Supplementary material for: Structural imbalances in EPA Perceptions: A multi-center study of cognitive differences between faculty and trainees in Chinese surgical residency training
Source: Surg Open Sci. 2026 Jun 26;33:49–54. doi: 10.1016/j.sopen.2026.06.003 (PMC13330528; doi:10.1016/j.sopen.2026.06.003)
Supplement: Supplementary file 1 — Supplementary material [file mmc1.zip › renamed_bf9f5.pdf]

Content and Standards for Standardized Training of Resident Physicians (2022 Edition)

China Medical Doctor Association

August 2022

Preface

Standardized residency training serves as a pivotal initiative to advance healthcare reform and medical education reform, constituting an essential pathway for medical graduates to become qualified clinical physicians.By the end of 2013, seven government agencies including the former National Health and Family Planning Commission jointly issued the "Guidelines on Establishing a Standardized Resident Training System" (Guo Wei Ke Jiao Fa [2013] No.56), marking the official nationwide implementation of standardized residency training (hereinafter referred to as "residency training"). In August 2014, the former National Health and Family Planning Commission released the "Certification Standards for Standardized Resident Training Bases (Trial)" and "Training Content and Standards for Standardized Resident Training (Trial)" (collectively termed the "Two Standards"), establishing benchmarks for facility development and quality assurance that played a crucial role in advancing the residency training system.

With the deepening advancement of the residency training system, particularly the "Opinions of the General Office of the State Council on Deepening Medical-Education Synergy and Further Promoting Medical Education Reform and Development" (State Council Document No.63 [2017]) and the "Guiding Opinions of the General Office of the State Council on Accelerating Innovative Development of Medical Education" (State Council Document No.34 [2020]), new and higher requirements have been proposed for improving the residency training system and enhancing the quality of talent cultivation. Meanwhile, due to changes in disease patterns, the widespread adoption and application of medical technologies, and the challenges posed by the COVID-19 pandemic, certain aspects of the original standards also require revision and refinement.

Commissioned by the Department of Science and Education of the National Health Commission, the China Medical Doctor Association (hereinafter referred to as the Association) initiated the revision of the "Two Standards" in August 2017. A "Guidance Group" was established, led by Academician Bian Xiuwu and composed of chairpersons of relevant postgraduate medical education professional committees and other experts, as well as an "Implementation Group" led by Geng Xiaobei, chairperson of the Training Quality Working Committee, and composed of members of the Training Quality Working Committee and experts from various professional committees. The two groups were responsible for revising the general provisions and specific details of each specialty, respectively. Over the past 4 years, a total of 616 management experts and clinical experts with extensive experience in medical education have participated in the revision of the "Two Standards."

During the revision process of the "Two Standards," the principles of "competency-oriented, problem-driven, pooling collective wisdom, and fact-based" were consistently adhered to. International advanced experiences and successful practices in residency training were referenced, and extensive feedback was solicited from experts across various professional fields, health administrative departments at all levels, relevant training bases, attending physicians, and trainees, resulting in over 3,000 feedback comments. The association organized experts to categorize and systematically analyze the feedback, with 610 reasonable suggestions being incorporated. The final version of the "Two Standards" (2022 edition) was thus formulated.

The main contents of this revision include: First, strengthening ideological and political education for resident physicians. The general principles of the new standard clearly state that resident training should be guided by Xi Jinping Thought on Socialism with Chinese Characteristics for a New Era, with moral education as the fundamental task, emphasizing the integration of ideological and political education throughout the entire resident training process. Second, it explicitly proposes six core competency requirements. The new standard draws on the achievements of the "China Elite Teaching Hospital Alliance" research on the "China Resident Physician Core Competency Framework" and international advanced experiences, adopting a competency-oriented approach and for the first time clearly defining training objectives as six core competencies.

The document outlines comprehensive requirements for core competencies and specifies detailed elements for each competency. Third, it emphasizes the enhancement of resident physicians' overall capabilities. The new standard categorizes training content into general knowledge and specialized knowledge, with strengthened training requirements in critical care medicine, emergency care, infectious diseases, and public health emergency response across internal medicine, emergency medicine, and general practice specialties. Fourth, it promotes a tiered progressive training model. Drawing on successful practices such as tiered progression recognized through eight years of training experience, all specialties have established clear tiered or phased training requirements, along with corresponding regulations on rotation schedules, subspecialty bed allocations, and disease classification criteria. Fifth, it improves operational standards for general practice training bases. It mandates that training bases located in general hospitals must independently establish general practice departments responsible for resident training programs. Clear requirements are also set for support mechanisms and incentive systems for general practice training. Sixth, it strengthens teaching organization and management. The primary responsible person at training bases must assume full accountability for resident training programs, while department directors serve as primary responsible persons for training quality across their specialized bases (including collaborating institutions). Training bases are required to appoint dedicated administrative staff, establish teaching clinics and clinical teaching beds, and implement integrated management systems for all specialized bases and partner institutions. Seventh, ensuring reasonable compensation for resident physicians. Training bases are explicitly required to guarantee resident physicians' authority and qualifications to conduct clinical work during training, establish salary and benefit standards, and provide reasonable compensation in accordance with regulations. Eighth, unified requirements are imposed on base capacity and its calculation methods. Specific methods for calculating capacity are defined for each specialty, with strict minimum training capacity requirements established based on professional characteristics.

"The Two Standards" (2022 Edition) embodies the dedication and hard work of health authorities, residency training management officials, and numerous medical education experts. Here, the association extends its highest respect to all experts who worked tirelessly day and night on the revision process! We also express our heartfelt gratitude to the "China Elite Teaching Hospital Alliance" and all colleagues who contributed suggestions for the standard revision!

China Medical Doctor Association
August 5, 2022

Catalogue

general rules······(1)

1. Detailed Rules for Internal Medicine Training······(5)
2. Pediatric Training Guidelines······(23)
3. Emergency Department Training Guidelines······(34)
4. Dermatology Training Guidelines······(55)
5. Detailed Guidelines for Psychiatric Training······(67)
6. Training Guidelines for Neurology Department······(76)
7. General Practice Training Guidelines······(84)
8. Training Guidelines for the Department of Rehabilitation Medicine······(124)
9. Detailed Training Guidelines for the Intensive Care Medicine Department······(132)
10. Detailed Guidelines for Surgical Training······(146)
11. Detailed Training Guidelines for Surgery (Neurosurgery Specialization)······(156)
12. Detailed Training Guidelines for Surgery (Thoracic and Cardiothoracic Surgery Specialty)······(168)
13. Detailed Training Guidelines for Surgery (Urology Specialty)······(178)
14. Detailed Training Guidelines for Surgery (Orthopedic Surgery Specialization)······(191)
15. Orthopedic Training Guidelines······(202)
16. Pediatric Surgery Training Guidelines······(221)
17. Detailed Guidelines for Obstetrics and Gynecology Training······(235)
18. Detailed Guidelines for Ophthalmology Training······(246)
19. Training Guidelines for Otorhinolaryngology······(254)
20. Anesthesiology Training Guidelines······(263)
21. Detailed Training Guidelines for Clinical Pathology Department······(271)
22. Training Guidelines for the Department of Laboratory Medicine······(279)
23. Radiology Department Training Guidelines······(298)
24. Training Guidelines for the Department of Ultrasound Medicine······(312)
25. Training Guidelines for Nuclear Medicine Department······(320)
26. Training Guidelines for Radiation Oncology Department······(329)
27. Training Guidelines for Medical Genetics Department······(340)
28. Detailed Guidelines for General Dentistry Training······(351)

29. Training Guidelines for Oral Medicine Department…………………………… (365)
30. Detailed Training Guidelines for Oral and Maxillofacial Surgery……………… (381)
31. Training Guidelines for Oral Rehabilitation Department…………………………(392)
32. Detailed Training Guidelines for Orthodontics Department…………………… (402)
33. Detailed Training Guidelines for Oral Pathology Department……………………(411)
34. Detailed Training Guidelines for Oral and Maxillofacial Radiology Department… (422)
Appendix: Editorial Board………………………………………………………… (432)

General Rules

In accordance with the requirements of the "Opinions of the General Office of the State Council on Deepening Medical-Education Synergy and Further Promoting the Reform and Development of Medical Education" (Guo Ban Fa [2017] No.63), the "Guiding Opinions of the General Office of the State Council on Accelerating the Innovative Development of Medical Education" (Guo Ban Fa [2020] No.34), the "Guiding Opinions on Establishing a Standardized Training System for Resident Physicians" (Guo Wei Ke Jiao Fa [2013] No.56), and the "Administrative Measures for Standardized Training of Resident Physicians (Trial)" (Guo Wei Ke Jiao Fa [2014] 49), this standard is promulgated to ensure the quality of standardized training for resident physicians and better meet the health and wellness requirements of the people and China.

I. Training Objectives

Comprehensively implement the fundamental task of fostering virtue and nurturing talent, cultivating clinical physicians with excellent professional ethics and expertise, who possess strong ideological integrity, professional competence, and work style, and are capable of independently and standardly performing diagnosis and treatment of common and frequently encountered diseases in their specialty. The core competencies are primarily reflected in the following six aspects.

(1) Professional Competence

Devoted to the motherland and passionate about the medical profession, adhering to the professional ethos of respecting and protecting life, rescuing the dying and healing the wounded, willingly making sacrifices, and boundless compassion, while upholding humanitarian professional principles; complying with laws and industry regulations, practicing self-discipline and self-respect, and maintaining integrity and trustworthiness.

Possess compassion, sense of responsibility, and altruistic spirit, adhere to the patient-centered medical philosophy, respect and safeguard patients' rights and interests, and protect patient privacy; be familiar with the healthcare system and related policies, regulations, and procedures, adept at identifying areas of deficiency, and propose improvement suggestions.

(II) Professional Competence

Possess foundational knowledge in basic medicine, clinical medicine, preventive medicine, as well as humanities and law, and be capable of applying it to healthcare practice; understand the national healthcare service system, medical insurance system, and medical education system; be familiar with the basic situation and latest developments in healthcare system reform.

Standardize and effectively collect patients' medical information, integrate and summarize various data to establish comprehensive analytical foundations; master diagnostic methods and formulate evidence-based clinical judgments; cultivate evidence-based medical thinking, adhere to professional guidelines and optimal evidence, while balancing clinical experience and patient needs to make informed diagnostic and therapeutic decisions; acquire essential clinical skills through training in the diagnosis, treatment, and management of common and frequently encountered diseases, thereby developing the capacity for independent practice in this specialty.

(III) Patient Management

With patient medical safety as the core principle, professional expertise is utilized to meticulously monitor disease progression, rationally prioritize treatment interventions, develop individualized clinical management plans, and provide effective and appropriate healthcare services.

serve 。

(IV) Communication and Cooperation

Possess clinical communication skills with strong humanistic awareness, apply principles and methods of doctor-patient communication to demonstrate appropriate empathy and establish a trusting and harmonious doctor-patient relationship; effectively obtain patient condition information or convey medical information to patients (families); respect individual needs of patients (families) and achieve collaborative decision-making between physicians and patients through thorough communication.

Maintain timely and effective communication and collaboration with the medical team; coordinate and utilize various available medical resources to address clinical practice challenges.

(5) Teaching Competence

Possessing teaching awareness, understanding commonly used clinical teaching methods, and participating in the guidance of medical students, junior resident physicians, and other healthcare professionals to collectively enhance professional competence, medical knowledge, and specialized skills; focusing on clinical practice to gradually develop clinical teaching capabilities.

be health promotion aware and use science popularization knowledge and skills to provide health behavior guidance to patients and the public.

(6) Learning Enhancement

Adopting the philosophy of autonomous learning and lifelong learning, actively utilizing various academic resources, and continuously engaging in self-reflection and improvement; persistently tracking medical advancements to update medical knowledge and concepts; conducting or participating in scientific research work tailored to clinical issues and needs; formulating career development plans to achieve continuous self-perfection and enhance professional competencies.

II. Training Content

The standardized residency training program focuses on enhancing standardized clinical diagnostic and therapeutic capabilities, implemented by specialty. Centered on resident physicians and emphasizing six core competencies, the program involves learning and mastering the following content through clinical practice under the guidance of senior physicians:

(1) General Education Content (Including Public Courses)

Master the content of ideological and political education and integrate it into value shaping and competency development; Understand health-related laws, regulations, and rules such as the Basic Healthcare and Health Promotion Law of the People's Republic of China and the Physician Law of the People's Republic of China; Gain knowledge about China's basic healthcare service system and policies and progress in healthcare system reform; Be familiar with healthcare insurance and medical education policies; Acquire theoretical knowledge and practical principles related to public health, and embrace the concepts of comprehensive healthcare, holistic wellness, and universal health; Master fundamental knowledge and skills in the prevention and treatment of key and regional infectious diseases, as well as hospital infection control.

Master fundamental theories and commonly used techniques in medical humanities, medical ethics, and interpersonal communication; acquire knowledge and skills in clinical consultation, medical record documentation, clinical thinking and decision-making, as well as rational blood transfusion and rational drug use.

Familiarize with evidence-based medicine concepts, clinical teaching and research methods, enhance the study of medical professional foreign languages, improve personal comprehensive abilities, and lay a solid foundation for lifelong learning and career development.

(II) Professional Content (Including Professional Courses)

Professional knowledge acquisition should be guided by clinical needs, with a focus on clinical medical knowledge and skills specific to the discipline and related fields, and integrated throughout the entire process of clinical practice training.

Professional knowledge encompasses the etiology, pathogenesis, clinical manifestations, diagnosis and differential diagnosis, management approaches, and clinical pathways of common and frequently occurring diseases in the specialty and related disciplines. Professional skills include fundamental competencies relevant to the specialty, as well as the assessment and emergency resuscitation techniques for common critical conditions within the field.

III. Duration and Methods of Training

(1) Duration of Training

The standardized residency training period generally lasts for 3 years (i.e., 36 months). For full-time master's degree candidates in clinical medicine or stomatology who undergo clinical practice competency training in accordance with the requirements of standardized residency training, the actual duration of clinical practice training shall not be less than 33 months. Matters regarding reduction, extension, or withdrawal from training shall be implemented in accordance with relevant national regulations.

(II) Training Methods

1. Resident physicians complete their training tasks at standardized residency training bases. The training primarily involves rotations within their specialty and related departments. Resident physicians must promptly, thoroughly, and accurately document the actual training content completed during clinical training, and diligently fill out the "Resident Physician Standardized Training Registration Handbook" with factual accuracy.

2. Centered on six core competency requirements and following the principle of "progressive implementation by year or phase," clinical practice, theoretical learning, and teaching activities are conducted to ensure that resident physicians progressively complete rotations in their specialty and related departments in accordance with the training guidelines, thereby meeting the training requirements.

3. Clinical practice should primarily focus on bedside patient management and/or outpatient practice; theoretical learning can be conducted through centralized face-to-face instruction, distance education, and planned self-study; teaching activities may employ various formats such as teaching rounds, outpatient teaching, clinical mini-lectures, case discussion sessions, and simulated teaching.

IV. Training Assessment

Training assessment comprises process evaluation and final evaluation. Process evaluation primarily includes daily assessments, departmental evaluations, annual evaluations, and annual competency tests. The assessment content should cover medical ethics, professional competence, attendance records, theoretical knowledge, clinical practice skills, training completion status, participation in teaching and professional development activities, with emphasis on comprehensive and systematic evaluation of core competencies in resident physicians. Assessment formats may include theoretical examinations and clinical practice assessments suitable for implementation at training sites.

Only those who pass the process evaluation and the National Physician Qualification Examination are eligible to participate in the standardized residency training completion assessment. The completion assessment includes theoretical evaluation and clinical practice competency assessment. Only those who pass both components will be awarded the "Standardized Residency Training Completion Certificate" supervised by the National Health Commission.

V. Others

(1) Each specialty shall comply with the requirements of these General Principles and implement the work in accordance with the corresponding professional training guidelines.

(2) Provinces (autonomous regions and municipalities) may appropriately adjust the content of relevant professional training based on their local disease spectrum, provided that it does not fall below the requirements specified in the corresponding professional training guidelines, and shall file a report with the China Medical Doctor Association.

(3) The content and standards for standardized training of resident physicians in Traditional Chinese Medicine (TCM) categories shall be formulated separately by the National Administration of Traditional Chinese Medicine and Pharmacy.
